# The case for development of a core outcome set (COS) and supplemental reporting guidelines for influenza vaccine challenge trial research in swine

**DOI:** 10.3389/fvets.2025.1465926

**Published:** 2025-02-11

**Authors:** Sheila Keay, Famke Alberts, Annette M. O’Connor, Robert Friendship, Terri O’Sullivan, Zvonimir Poljak

**Affiliations:** ^1^Department of Population Medicine, Ontario Veterinary College, University of Guelph, Guelph, ON, Canada; ^2^Department of Large Animal Clinical Sciences, College of Veterinary Medicine, Michigan State University, East Lansing, MI, United States

**Keywords:** core outcome set (COS), evidence-based medicine (MeSH), IAV-S vaccine, reporting guideline adherence, swine, research waste, influenza A viruses of swine

## Abstract

Previously, we systematically reviewed more than 20 years of influenza vaccine challenge trial research in pigs to answer the question, “does vaccinating sows protect offspring?” Overall, most studies were well designed but clinical heterogeneity made between-study comparisons challenging. Studies varied by samples, outcomes, and assays selected for measurement. Additionally, data essential for inclusion of findings in meta-analyses were often insufficiently reported and as a result, summary effect measures were either not derived or were not meaningful. Clinical heterogeneity and reporting issues complicate and limit what can be learned cumulatively from research and both represent two types of avoidable research waste. Here, we illustrate each concern using data collected tangentially during the systematic review and propose two corrective strategies, both of which have broad applicability across veterinary intervention research; (i) develop a Core Outcome Set (COS) to reduce unnecessary clinical heterogeneity in future research and (ii) encourage funders and journal editors to require submitted research protocols and manuscripts adhere to established reporting guidelines. As a reporting corollary, we developed a supplemental checklist specific to influenza vaccine challenge trial research in swine and propose that it is completed by researchers and included with all study protocol and manuscript submissions. The checklist serves two purposes: as a reminder of details essential to report for inclusion of findings in meta-analyses and sub-group meta-analyses (e.g., antigenic or genomic descriptions of influenza vaccine and challenge viruses), and as an aid to help synthesis researchers fully characterize and comprehensively include studies in reviews.

## 1 Introduction—avoidable research waste and corrective strategies

In 2014, evidence-based medicine (EBM) methodologists authored a series of seminal articles in the journal, *The Lancet*, describing five areas of avoidable waste in biomedical research and offered corrective strategies for each (1–5). Multiple stakeholders use research for decision making but recognizing outcomes most relevant to users were not consistently selected, measured, or reported in research, methodologists also focused on standardization of endpoints (6, 7). Products of this effort include:

2010 – development of the COSMIN checklist (Consensus-based Standards for the selection of health status Measurement INstruments) as an aid for evaluating the methodological quality of studies investigating measurement properties (8, 9).

- 2014 – formation of COMET, the Core Outcome Measures in Effectiveness Trials initiative (9).
- 2016 – establishing COS-STAR, Core Outcome Set-STAndards for Reporting as reporting guidelines for COS development studies (10, 11).
- 2017 – establishing COS-STAD, Core Outcome Set-STAndards for Development to outline minimum standards for the design of COS development studies (12).
- 2019 – providing COS-STAP, Core Outcome Set-STAndardized Protocol Items, a checklist of 13 items considered essential documentation in protocols for COS development studies (13).

Core outcome sets (COS) are adopted to harmonize inclusion of essential elements of study design in clinical research (2, 11). A COS is “an agreed minimum set of outcomes that should be measured and reported in all clinical trials of a specific disease or trial population”; it is disease or population specific but it is not trial specific, meaning it is a recommendation of *what* should be minimally measured and reported in all clinical trials, but not *how* (9, 14, 15). Establishment of core outcome sets (COS) address issues of research waste associated with avoidable differences from one study to the next in the elements of study design (i.e., clinical heterogeneity) (2, 12).

Adoption of a COS approach reduces waste and improves the value of research to users in three ways; (1) it ensures outcomes most relevant to stakeholders are included in research, (2) it reduces outcome reporting bias (i.e., prevents selective reporting of only a sub-set of measured outcomes), and (3) it ensures all trials contribute usable information in meta-analyses (14). Inclusion of each study’s data in meta-analyses means that resources invested in primary research may be further leveraged through synthesis (16–18). Hundreds of core outcome sets (COS) have been developed in human healthcare (19) but we are aware of only two COS initiatives in veterinary care; COSCAD’18 for atopic dermatitis in dogs (20) and development of a COS for feline chronic kidney disease treatment trials (21). However, awareness is growing and recently, Sargeant et al. (22) described how a COS approach maximizes the utility of intervention research trials conducted in swine populations.

Within the context of *The Lancet* series (2, 5), we revisit our systematic review and meta-analysis of 20 years of influenza challenge trial research in swine (published between 1990 and May 2021) (23) to explore and illustrate two types of avoidable waste found in that body of evidence. Our perspective for this work was gained through prior investigation of the barriers that slow translation of influenza research into useful knowledge for swine practitioners (23–25). We use data collected tangentially during the systematic review to identify clinical heterogeneity and reporting insufficiencies and based on this, propose two corrective strategies; development of a COS using standard and established Delphi consensus building processes (12), and, in addition to promoting use of established reporting guidelines, propose adoption of a novel supplemental reporting checklist specific to influenza vaccine challenge trial research in swine.

## 2 Clinical heterogeneity and reporting insufficiency in swine MDI challenge trial research

The systematic review was conducted to answer the question of whether the common industry practice of vaccinating sows against influenza conferred protection (via maternally derived immunity (MDI)) to their offspring (23). Data on six different outcomes were characterized and extracted for meta-analyses. Outcomes included three direct measures of infection, a gold standard immune correlate of protection (CoP), and two clinical signs. Measures of effect (also known as treatment measures or effect sizes) were impacted by the match between vaccine antigens and corresponding challenge virus(es), but overall, challenge trial evidence neither supported nor refuted vaccination of sows to protect piglets (23).

*Post hoc*, we found the strength of the systematic review and specifically, confidence in summary effect measures, was reduced due clinical heterogeneity, and due to incomplete reporting of trial information and data. Reporting was not necessarily incomplete from the standpoint of communicating finding of individual studies, but rather, details were frequently omitted that were essential for inclusion in meta-analyses (i.e., measures of centrality, measures of dispersion, group sizes) and for inclusion in sub-group meta-analyses (i.e., antigenic characterization of vaccine and challenge viruses) (23). Table 1 is a summary of the number of studies that included one or more of the six outcomes versus the number of studies that were included in the meta-analysis (MA) of each outcome. Proportionately, few eligible studies were included in meta-analyses.

**Table 1:** Number of studies by reported outcomes versus studies reporting sufficient data for inclusion in meta-analysis (*N* = 15^a^ influenza challenge studies in piglets with vaccine-derived MDI published between 1990 and 2021, and reporting at least 1 of 6 eligible outcomes).

Outcome	No. of studies measuring outcome	No. included in MA	MA effect measure
Direct measures^b^:			
Virus detection	14	5	RR
Starting to shed virus over study period	13	4	HR
Ceasing to shed virus over study period	13	4	HR
Time to start shedding virus	13	4	MD
Duration of virus shedding (time to stop)	13	4	MD
Virus quantity	12	7	SMD
Indirect measures:			
HAI	13	2	MD
ADG	3	2	MD
Coughing	5	1	RR

a Sixteen studies were eligible for characterization in the systematic review. Fifteen reported outcomes eligible for inclusion in meta-analysis. Data was collected tangentially during the systematic review of IAV-S influenza challenge trials in piglets with maternally derived immunity (MDI) from vaccinated sows (*N* = 16). MDI, maternally derived immunity; MA, meta-analysis; effect measures (a.k.a. treatment effect/effect size) were either extracted as reported in primary research or were calculated from reported data.

b Derived from nasal swab samples only (i.e., excludes data from lung and nasal wash samples); RR, relative risk; HR, hazard ratio; SMD, standardized mean difference; MD, mean difference; ADG, average daily gain; HAI, hemagglutination inhibition assay.

### 2.1 Clinical heterogeneity and elements of study design

To illustrate clinical heterogeneity we looked at three elements of study design; (i) outcomes measured, (ii) samples collected, and (iii) assays employed, then further characterized elements into sub-elements and identified use of each across all studies. Overall, the body of research was complex applying different combinations of samples collected, assays used, and outcomes measured, both within and across studies.

### 2.2 Clinical heterogeneity and network analysis

**Figure 1:**
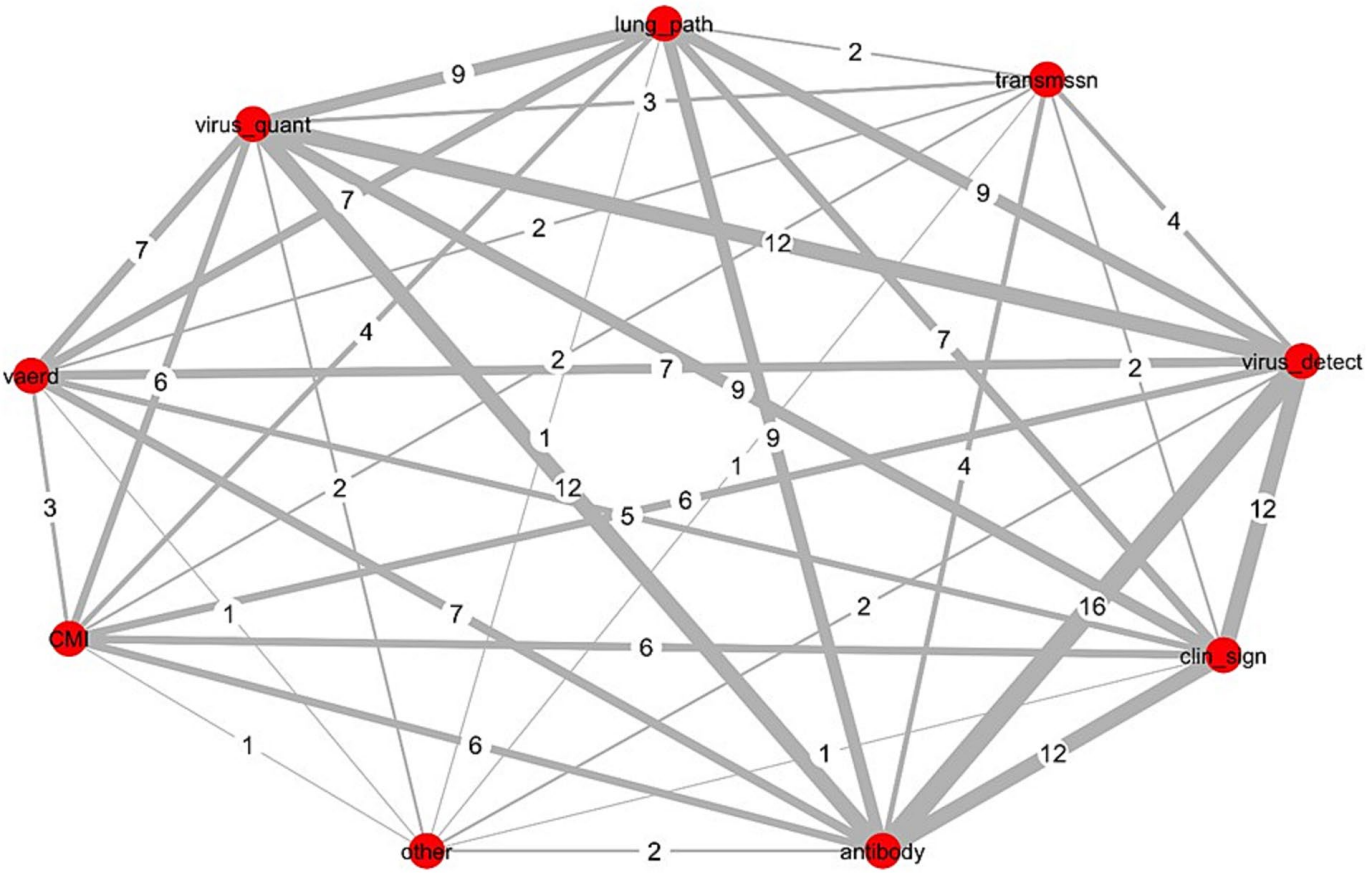
Network analysis of outcomes measured across 16 influenza challenge trials in piglets with vaccine-derived maternal immunity. Data was collected tangentially during the systematic review of IAV-S influenza challenge trials in piglets with maternally derived immunity (MDI) from vaccinated sows (*N* = 16) (23); Red circles (nodes), outcome measured; numbers assigned each grey line (edge), number of studies jointly reporting both outcomes (thickness of line increases with increased frequency of joint reporting); CMI, all non-immunoglobulin immune responses including cell mediated immunity; vaerd, vaccine associated enhanced respiratory disease; Virus_quant, virus titres extrapolated from PCR measurements or from virus isolation methods; lung_path, observed macro or microscopic evaluation of lung lesions; transmissn, virus transmission from an infected pig to an uninfected pig; virus_detect, virus detection via RT-PCR or virus isolation; clin_sign, clinical signs; antibody, immunoglobulin titres; other, microtracheal lesions.

We used methods of network analyses to generate displays showing the scope and relatedness of each of the three elements as applied across all studies (*N* = 16). Figure 1 and Supplementary Figures S1, S2, display the number of studies jointly including the same sub-elements of outcomes measured, samples collected, and assays used across all studies. The Delphi method for reaching consensus is a preferred approach for developing a COS and this type of visual can, at the onset of the process, help Delphi study participants to understand the extent of existing clinical heterogeneity (9, 12, 21, 26).

#### 2.2.1 Network analysis of outcomes measured

Outcomes were categorized into 9 sub-elements. Figure 1 shows the connectivity of each across all 16 studies. Nodes (red circles) represent the sub-element, and the edges (connecting lines) equal the number of studies jointly measuring both sub-elements. Virus detection and antibody titres were measured in all studies; each was most frequently paired with clinical signs (*n* = 12) and virus quantification (*n* = 12). Other frequent pairings included lung pathology with each of virus detection (*n* = 9), virus quantification (*n* = 9), and antibody measures (*n* = 9). Virus quantification and clinical signs were jointly measured across more than half of all studies (*n* = 9).

#### 2.2.2 Network analysis of samples collected

Samples were categorized into 17 sub-elements (see Supplementary Figure S1). Multiple sub-elements were collected in each study and the most frequent pairing of samples across studies was nasal swabs with serum (*n* = 14), both of which were also paired frequently with dyspnea (*n* = 10). Next most frequent pairings were fever with each of serum (*n* = 12), nasal swabs (*n* = 11), or dyspnea (*n* = 10). Half of all studies (*n* = 8) included both collection of serum and bronchioalveolar lavage fluids (collected post mortem).

#### 2.2.3 Network analysis of assays used

Assays were categorized into 15 sub-elements (see Supplementary Figure S2); most frequent pairings were ELISA assays (ELISA_2) with cell culture (for virus isolation) (*n* = 13), followed by ELISAs with measure of hemagglutination inhibition titres (HAI) (*n* = 12), and HAI with cell culture (*n* = 10). Half of all studies included measures of macroscopic lung lesions (L_macro) with each of lung histological lesions (L_micro) (*n* = 8), HAI (*n* = 9), immunoglobulins (ELISA_2) (*n* = 8), or cell culture (*n* = 8). All microscopic reporting of lung lesions (L_micro) was paired with (HAI) (*n* = 8).

### 2.3 Summary of the clinical elements of study design across all studies (*N* = 16)

All studies included as outcomes measures of immunoglobulin responses and of virus detection; inclusion of other outcomes was variable (see Table 1). Samples collected and assays employed to measure each of the six outcomes varied across all 16 studies and are summarized in Supplementary Figures S3, S4, respectively.

Nasal swabs were used to collect samples for virus detection in 14/16 studies (Supplementary Figure S3). Three approaches were taken to demonstrate direct evidence of virus in piglets post challenge (Supplementary Figure S4); (1) culture of live virus (*n* = 13), (2) detection of viral RNA nucleic acids using polymerase chain reaction (PCR) methods (*n* = 6), and (3) histological identification *in situ* of viral antigen in sectioned tissues [immunohistochemistry (IHC)] (*n* = 5). Virus was quantified using cell culture methods (*n* = 10) and RT-qPCR methods (*n* = 4).

Serum immunoglobulin titres were measured in all but 1 study using enzyme linked immunosorbent assays (ELISA) (*n* = 15) (Supplementary Figure S4). ELISAs also varied with respect to the type of immunoglobulins measured; as IgG and /or IgA isotypes or as immunoglobulins specifically against conserved viral proteins NP (nucleoprotein) or M (matrix protein). The HAI assay, which is the gold standard correlate of protection, was used in most studies (*n* = 13) to identify sub-type and strain specific immunologic responses to influenza A viruses of swine.

Although few studies included outcomes measuring cell mediated immune responses (CMI), the assays used were varied; multiparameter flow cytometry (*n* = 6) was used to assess T-cell proliferation (staining for detection of CD4+, CD8+, CD3+, γδ TCR+ peripheral blood mononuclear (PBMN) cell populations) and T-cell priming (staining for CD25, IFN-gamma, and IL-10) (Supplementary Figure S4). ELISA based assays including the enzyme-linked immunospot (ELISpot) assay (*n* = 1), used for detection of IFN-gamma secreting cells, and a multiplex ELISA (*n* = 2) used to detect cytokine production. Clinical signs were measured as a stand-alone measure or as part of a composite score in 12 of 16 studies and are summarized in Supplementary Figure S5. Fever was the most consistently measured and reported clinical sign.

### 2.4 Supplemental reporting of influenza vaccine challenge trials in swine

Incomplete reporting of study methods and results hinders assessment for internal and external validity (27, 28), and in veterinary medicine is a cause of frequent exclusion of primary research studies from systematic reviews and meta-analyses (18, 29–34). In our review, studies were excluded from sub-group meta-analyses if hemagglutinin and neuraminidase antigens in vaccines were poorly characterized (23). This further diminished the value of inferences derived through synthesis. Therefore, we encourage funders and journal editors require research protocols and manuscripts adhere to established reporting guidelines such as REFLECT (available on the Meridian Network at https://meridian-network.org/).

Additionally, we developed a reporting checklist specific to influenza vaccine challenge trails in swine (see Supplementary Tables S1, S2) and encourage researchers to complete and include the checklist as a supplemental document with all manuscript submissions. The checklist itemizes sub-elements of study design important for overall contextual understanding of influenza vaccine research (i.e., outcomes, samples and assays). The intent is that it serves as a reminder to researchers of essential items to report (35), and as an aid for synthesis researchers when identifying studies and extracting data for inclusion in systematic reviews and meta-analyses (31, 36). Consistent reporting of outcome, assay and sample sub-elements may also help to improve researcher, reviewer, and reader awareness of the full extent of avoidable clinical heterogeneity (8, 9).

## 3 Discussion

### 3.1 Objectives, endpoints, and definitions of influenza vaccine protection

Vaccine protection is a non-specific term and context is important for interpretation; protection from infection must be distinguished from protection against an undesired clinical endpoint (37) and no single outcome conveys protection against infection or disease (38). From a societal perspective, desired endpoints are conditioned on stakeholders’ need to inform decisions on implementation of vaccines in the field. For researchers involved in vaccine development work, desired endpoints are a function of the phase of research for which they are engaged (39, 40). Examples of researcher objectives, societal perspectives, and corresponding vaccine research endpoints and measures of protection against in influenza A viruses in swine (IAV-S) are listed in Supplementary Table S3.

In human influenza vaccine research, researchers’ objectives differ by each distinct phase of research (see Supplementary Table S3; Supplementary Figure S6) (38, 41–43). Phase “0″ is the proof-of-concept phase for candidate vaccines. In Phase I to III clinical trials, convenient methods and assays to measure efficacy are validated, safety, dosages, and schedules are established, and approval for market use is established. In Phase IV clinical trials, post marketing evaluation of field effectiveness is established. Compared to human vaccines, approval of veterinary vaccines is expedited due to:

- Fewer restrictions governing conduct of same species challenge trials (44–46).
- Veterinary vaccines are commonly approved based on Phase II challenge trial evidence (44).
- Post-marketing evaluation of vaccine performance is not required (44).
- A regulatory framework exists for restricted market use of non-licensed autogenous vaccines without proof of efficacy (46).

Desired endpoints overlap in early phase and late phases of vaccine research (Supplementary Table S3) (38). Validating correlates of protection (CoP), specifically immune CoPs, is often done using challenge trials in animal models, where same-species challenge models are optimal (47–49). Historically, hemagglutination inhibition (HAI) serologic assays have been the gold standard CoP for evaluating efficacy of inactivated vaccines against influenza A virus (IAV) (50), however because understanding of immune responses to influenza is incomplete, multiple outcomes must be assessed to accurately predict vaccine performance (37, 51). Additionally, use of live vaccines and newer vaccine technologies means both cell-mediated CoPs and direct measures of virus infection are needed to assess protection (50, 52–54).

There are gaps in understanding of how a host’s immune responses are coordinated to eliminate virus following primary infections, and of the impact of prior or original virus exposure on immunologic responses to secondary strain-homologous and to strain-heterologous IAV exposures (55). Universal vaccines and novel vaccine platforms typically elicit immunologic responses to non-dominate epitopes (i.e., the dominant HA receptor binding sites are not targeted) and as emphasis shifts to the design of such vaccines, so too will study of unintended adverse outcomes such as vaccine enhanced acute respiratory disease (VEARD) (56–61).

### 3.2 The role of challenge trials and swine influenza research in vaccine development

Although rare in human medicine (45, 62), challenge trials were the most frequently reported study design in IAV-S vaccine research in swine published since 1990 (25). For each of the 16 MDI challenge studies, the author’s stated objective(s) and study endpoints are summarized in Supplementary Table S4, indicating also where objectives correspond to one or more of the five critical R&D immunologic issues outlined in The Influenza Vaccine Research and Development (R&D) Roadmap (51).

Within a One Health context., IAV-S vaccine challenge research in swine contributes to the larger influenza research community of practice, and in this light, development of a COS for IAV-S research in swine (using a Delphi process) may serve as a template for development of COS for influenza vaccine research in other animal species (63, 64). Similarly, adoption of the proposed checklist may also serve as a multi-species template for consistent reporting of essential elements important for research synthesis and ultimately for the interpretation of vaccine challenge trial studies in animals.

## 4 Conclusion

Within the context of avoidable research waste, we illustrated how clinical heterogeneity and reporting insufficiencies led to substantive exclusion of IAV-S MDI challenge trials in swine from subsequent qualitative and quantitative synthesis of the body of research evidence. We advanced two corrective actions; development of a core outcome set (COS) using Delphi consensus building methods, and in addition to encouraging adherence to established reporting guidelines, use of a novel supplemental reporting checklist specific to influenza challenge trials in swine. We suggested a completed checklist accompanies all primary research manuscripts as an aid to funders, editorial reviewers, readers, and synthesis researchers, for improving contextual understanding, and to facilitate charting and extracting IAV-S specific details during synthesis.

## Data Availability

The original contributions presented in the study are included in the article/Supplementary material, further inquiries can be directed to the corresponding author.
