# Supplementary material for: The case for development of a core outcome set (COS) and supplemental reporting guidelines for influenza vaccine challenge trial research in swine
Source: Front Vet Sci. 2025 Feb 11;12:1465926. doi: 10.3389/fvets.2025.1465926 (PMC11851948; doi:10.3389/fvets.2025.1465926)
Supplement: Supplementary file 1 [file Data_Sheet_1.docx]

***Supplementary Material***

**The case for development of a core outcome set (COS) and supplemental reporting guidelines for influenza vaccine challenge trial research in swine**

**Sheila Keay†, Famke Alberts, Annette M. O’Connor, Robert Friendship, Terri O’Sullivan, and Zvonimir Poljak**

**Correspondence:** Sheila Keay: [sheilakeay@gmail.com](mailto:sheilakeay@gmail.com)

**Supplementary Table 1: Proposed IAV-S vaccine research reporting checklist (pg. 1 of 2).**

| **Study number / study identifier** (complete for each study reported in publication).   \| *Enter identifier* \| \| --- \|   Select all that apply, enter text as indicated **Population IAV-S exposure history (prior to vaccination)**   \|  \| Unknown \| \| --- \| --- \| \|  \| Naïve \| \|  \| Known, not source of vaccine seedstock \| \|  \| Known, source of vaccine seedstock \| \|  \| Maternal derived immunity - known sow exposure \| \|  \| Maternal derived immunity -unknown sow exposure \|   Provide phenotypic/ genotypic description of all **pre-**vaccination antigenic exposure   \| *Enter text* \| \| \| \| \| --- \| --- \| --- \| --- \| \| **Vaccine type applied** \| \| **Vaccine valency** \| \| \| \|  \| Experimental \|  \| Monovalent \| \| \|  \| Commercial general market \|  \| Multivalent \| \| \|  \| Commercial autogenous \|  \| Not identified \| \|   List commercial manufacturer/ brand name(s)   \| *Enter text* \| \| \| \| \| --- \| --- \| --- \| --- \| \| **Vaccine platform** \| \| **Age at vaccination** \| \| \| \| Non-replicative \| \|  \| Pre-weaning \| \| \|  \| Whole inactivated killed \|  \| Growing pig \| \| \|  \| Split virus \|  \| Reproductive female \| \| \|  \| Sub-unit \|  \| Reproductive male \| \| \|  \| plasmid DNA \|  \|  \| \| \|  \| RNA / replicon particle \|  \|  \| \| \|  \| Virus-like particles (VLP) \|  \|  \| \| \| Replicative \| \|  \|  \| \| \|  \| Live attenuated influenza virus \|  \|  \| \| \|  \| Viral vector \|  \|  \| \| \| **Vaccine antigenic description** \| \|  \|  \| \| \|  \| Commercial product - not available \|  \|  \| \|   Provide available vaccine phenotypic, genotypic description   \| *Enter text* \| \| \| \| \| --- \| --- \| --- \| --- \| \| **Post-vaccination virus exposure** \| \| **Exposure type** \| \| \| \|  \| Yes \|  \| Natural \| \| \|  \| No \|  \| Challenge trial \| \| \| **Challenge frequency:** \| \|  \|  \| \| \|  \| Single \|  \|  \| \| \|  \| Repeated same strain \|  \|  \| \| \|  \| Repeated different strain(s) \|  \|  \| \| \|  \| Multiple strain simultaneous \|  \|  \| \|   Provide phenotypic, genotypic description of all post-vaccination virus exposure   \| *Enter text* \| \| --- \|   . |
| --- | --- | --- | --- | --- | --- | --- | --- | --- | --- | --- | --- | --- | --- | --- | --- | --- | --- | --- | --- | --- | --- | --- | --- | --- | --- | --- | --- | --- | --- | --- | --- | --- | --- | --- | --- | --- | --- | --- | --- | --- | --- | --- | --- | --- | --- | --- | --- | --- | --- | --- | --- | --- | --- | --- | --- | --- | --- | --- | --- | --- | --- | --- | --- | --- | --- | --- | --- | --- | --- | --- | --- | --- | --- | --- | --- | --- | --- | --- | --- | --- | --- | --- | --- | --- | --- | --- | --- | --- | --- | --- | --- | --- | --- | --- | --- | --- | --- | --- | --- | --- | --- | --- | --- | --- | --- | --- | --- | --- | --- | --- | --- | --- | --- | --- | --- | --- | --- | --- | --- | --- | --- | --- | --- | --- | --- | --- | --- | --- | --- | --- | --- | --- | --- | --- | --- | --- | --- | --- | --- | --- | --- | --- | --- | --- | --- | --- | --- | --- | --- | --- | --- |

**Supplementary Table 2. Proposed IAV-S vaccine research reporting checklist (pg. 2 of 2).**

| Select all that apply | | |  | | |  | | |
| --- | --- | --- | --- | --- | --- | --- | --- | --- |
| **Outcomes measured** | | | **Assay types employed** | | | **Sample types collected** | | |
|  | Lung lesions | |  | Lung lesions = macroscopic | |  | Serum | |
|  | CMI† | |  | Lung lesions = microscopic | |  | Nasal swabs | |
|  | Immunoglobulins | |  | Basic reproductive number (R^0^) | |  | Udder wipes | |
|  | Virus detection | |  | Multiplex ELISA for cytokines | |  | Nasal wipes | |
|  | Virus quantification | |  | ELISpot | |  | BALF | |
|  | Time to shedding virus | |  | Flow cytometry | |  | PBMN cells | |
|  | Duration of virus shedding | |  | ELISA - immunoglobulins | |  | Lung tissue -gross | |
|  | Transmission | |  | HAI | |  | Lung tissue - fixed | |
|  | VAERD | |  | ELISA IgA | |  | Nasal wash | |
|  | Fever | |  | ELISA IgG | |  | TBLN | |
|  | Dyspnea | |  | Virus neutralization | |  | Milk | |
|  | Lethargy | |  | Virus isolation from cell culture | |  | Colostrum | |
|  | Coughing | |  | Immunohistochemistry | |  | Environmental- surface | |
|  | ADG | |  | RT_PCR | |  | Environmental- Air | |
|  | Mortality | |  | RT_qPCR | |  |  | |
|  | Sneezing | |  |  | |  |  | |
|  | Other: |  |  | Other: |  |  | Other: |  |
|  | *Enter text* |  |  | *Enter text* |  |  | *Enter text* |  |

†CMI = all non-immunoglobulin immune response including cell mediated immunity;

ELISpot = ELISA based assay for IFN-gamma secreting cells; ELISA- immunoglobulins = detection of non-isotype specified immunoglobulins; ELISA IgG = detection of isotype IgG anti-IAV-S immunoglobulins; ELISA IgA = detection of isotype IgA anti-IAV-S immunoglobulins; HAI - hemagglutination inhibition; Flow cytometry = Multi-parameter flow cytometer (T-cell proliferation assay measuring staining for CD4+, CD8+, CD3+, γδ TCR+ PBMN cell populations (proliferation), and/ or for measuring T-cell priming up-regulation of T-cell markers (e.g. CD25, IFN-gamma, IL-10)); PBMN = peripheral blood mononuclear cells; Antibody = immunoglobulin immune response; Virus detection = identification of virus present via RT-PCR or virus isolation; virus quantification = virus titres from samples using PCR or virus isolation methods; lung lesion = macro/ microscopic evaluation; CMI = all non-immunoglobulin immune response including cell mediated immunity; VAERD = vaccine associated enhanced respiratory disease an outcome determined based on measure of lung pathology and clinical presentation of respiratory distress; PBMN = peripheral blood mononuclear cells (from whole blood); BALF = bronchoalveolar lavage fluids; TBLN = tracheobronchial lymph nodes.

**Supplementary Table 3. Researcher objectives / societal perspectives by corresponding endpoints, and measures of vaccine protection**

| **Researcher Objectives** | **Priority end point** | **Measures of protection** |
| --- | --- | --- |
| Phase I | Identify correlates of immune protection (CoP) | Immunoglobulin (humoral) responses |
|  |  | Myeloid/lymphoid responses (identification of responding dendritic cells, monocytes, B-cells, T-cells) |
|  |  | Lymphoid cellular proliferation, up-regulation |
| Phase II/ II b (healthy experimental population) | Demonstrate immunogenicity  (validate CoPs) | Immunologic responses as per Phase I |
|  | Show no/minimal adverse reaction to vaccine | Clinical responses of vaccinees to vaccine (e.g. fever, pain, hypersensitivity) |
| (with exposure to pathogen) | Prevent infection | Incidence of infection, peak titres, duration of shedding |
|  | Prevent clinical disease | Expression of clinical signs post-virus exposure (respiratory distress, fever, pain) |
|  | Demonstrate no adverse vaccine reactions following pathogen exposure | Enhanced severity of clinical signs in infected vaccinees versus infected non-vaccinees |
|  |  | (in animal challenge trials - post mortem pathological examinations e.g. lung lesions; in human trials - anti-mortem clinical diagnostics such as radiographs) |
|  | Measure viral resistance to vaccine | Infection/ clinical disease in vaccinees as compared to expected vaccine performance |
| Phase III (target population) | Prevent infection | Incidence of infection, peak titres, duration of shedding |
|  | Prevent clinical disease | Measures of CoPs validated in Phase II  Expression of clinical signs post-virus exposure (respiratory distress, fever, pain) |
|  | Demonstrate no adverse vaccine reactions post pathogen exposure | Enhanced severity of clinical signs in infected vaccinees versus infected non-vaccinees |
|  | Enable prediction of duration of immunity | Rate of antibody decline |
| **Societal Perspectives** | | |
| Licensure/ Regulatory | Efficient validation of vaccine efficacy, potency, purity, safety +/-environmental assessment | Measures of CoPs validated in Phase III  (measures of environmental impact – case by case as per vaccine platform type) |
| Phase IV – post marketing |  |  |
| Livestock health manager | Optimize production performance (demonstrate vaccine effectiveness) | Control = minimal disruption to production performance (various production metrics) |
|  | Eradication of infection | Incidence/prevalence of infection |
|  |  | Transmission |
| Health care provider | Prevent clinical disease | Hospitalizations, days in hospital |
|  | Reduction of societal disruption | Days off work/ school |
|  | (demonstrate vaccine effectiveness) | Disease incidence (laboratory confirmed) |
| Public Health Policy | Reduced zoonotic potential | Phenotype/ genotype changes† |
|  | Pandemic preparedness | Transmission (spread from infected to uninfected) |
|  | Control infection in population | Incidence/ prevalence of infection |
|  | Detection of viral resistance to vaccine  (evaluate vaccine effectiveness) | Increasing incidence/ prevalence/ severity of disease in vaccinees (laboratory confirmed) |

Table compiled from sources: Hudgens (2004), WHO (2004), CBER (2007), and CFIA (2022) [39,43,44,47]

† Synonymous/ non-synonymous nucleotide changes, segment reassortment events

**Supplementary Table 4. Matching of swine MDI study objectives and outcomes with IVR† identified critical R&D issues for influenza vaccine research** (Studies (N=16) ordered by date of publication)

| **Author*** | **Study objective summarized (*actual text from publication introduction*)** | **R&D issue 1** | **R&D issue 2** | **R&D issue 3** | **R&D issue 4** | **R&D issue 5** | **Researcher endpoint** |
| --- | --- | --- | --- | --- | --- | --- | --- |
| Kitikoon, 2006 | Efficacy of vaccination (commercial WIV) in piglets with MDI and assessment of immune response against heterologous challenge.  (*The research objectives were three-fold; the first objective was to investigate protection by MDA against experimental challenge with a heterologous SIV isolate; the second objective assessed vaccine efficacy against a heterologous SIV isolate in the absence of MDA; and the third objective examined the effect of MDA on vaccine efficacy in the piglets when experimentally challenged with a heterologous SIV*). | + | + | + |  | + | Prevent infection, prevent disease, show immunogenicity |
| Bosworth, 2010 | Efficacy of vaccination (HA RP vx) in piglets with MDI against homologous challenge.  (*The objectives of this study were to determine if (1) a RP vaccine derived from an alphavirus and expressing the HA gene could induce immunity and protection to homologous SIV in the absence of maternal antibody*). | + | + |  |  |  | Prevent infection, prevent disease |
| Vincent, 2012 | Efficacy and immunogenicity of vaccination (LAIV and WIV) in piglets with MDI against homologous and heterologous challenge.  (*In the present study, we tested the immunogenicity and protective efficacy of the intranasal NS1 126 TX98 vaccine versus an inactivated, adjuvanted TX98 vaccine administered intramuscularly in naive and MDA-positive weanling pigs subsequently challenged with homologous or heterologous strains of H3N2 IAV*). | + | + | + |  |  | Prevent disease, prevent infection, identify CoPs |
| Kitikoon, 2013 | Efficacy of vaccination (commercial WIV) in piglets with MDI against heterologous challenge.  (*A vaccine that induced the highest HI cross-reactivity to tested viruses against contemporary swine H3N2 viruses with genetically diverse H3 genes) was selected for further investigation to evaluate efficacy when delivered in the presence or absence of heterologous MDA*). | + | + | + |  |  | Prevent disease, prevent infection |
| Sandbulte, 2014 | Assessment of CoPs and efficacy of vaccination (LAIV, WIV) in piglets with MDI using a VAERD model with heterologous challenge.  (*Objectives of this study included testing the efficacy of a single dose LAIV regimen in piglets with and without MDA; determining the effects of MDA on cellular and mucosal immune responses; and identifying immune responses correlated with VAERD versus cross –protection upon Heterologous challenge*) | + | + | + |  | + | Prevent disease, identify CoPs, prevent infection |
| Loving, 2014 | Assessment of role of cellular component of MDI on outcomes in a VAERD model with homologous and heterologous challenge.  (*In the reported study, we used CF as an experimental approach to determine if MDC played a significant role in vaccine mis-priming that resulted in IAV related VAERD, or if the presence of only MDA alone was sufficient to drive the mis-priming response*). | + | + |  |  |  | Prevent disease, prevent infection, show immunogenicity |
| Pyo, 2015 | Efficacy of vaccination (LAIV) in piglets with MDI.  (*In this study, we explored whether vaccination of SIV/606 in piglets would overcome the MDA-associated obstacles investigating also effect of MDA on vaccine efficacy of LAIV delivered by systemic and local mucosal routes*) | + | + | + |  |  | Prevent disease, prevent infection |
| Rajao, 2016 | Investigation of VAERD model in MDI piglets with heterologous challenge. (*Here, we investigated if the presence of passive MDA at the time of heterologous challenge would result in enhanced disease in which seropositive sows previously naturally exposed to IAV were vaccinated with the same virus strain and their litters were challenged with the homologous or heterologous virus.)* |  | + | + |  | + | Prevent infection, prevent disease |
| Allerson, 2013 | Assessment of impact of MDI on IAV transmission with homologues and heterologous challenge.  (*The main objective of this study was to assess the impact of maternally derived immunity on IAV transmission by estimating and comparing the transmission rates, infectious periods, and reproduction ratios between groups of neonatal pigs with varying maternal immunity. In addition, the number of sentinel pigs infected per day and serum antibody titers were compared between treatment groups*). |  | + |  |  |  | Prevent infection, prevent disease |
| Cador, 2016 | Assessment of impact of MDI on duration of virus shedding and transmission in piglets.  (*The aim of this study was therefore to clarify the impact of MDAs on the dynamics of infection in young piglets both in terms of transmission (direct and indirect) and duration, by quantifying and comparing swIAV spread in piglets in the presence and absence of MDAs under experimental conditions*). |  | + |  |  |  | Efficacy, prevent infection, prevent clinical disease |
| Deblanc, 2018 | Assessment of MDI on piglet immune response, performance, and virus shedding after first and second challenge at different piglet ages (…*performed an experimental study with MDA- and MDA+ animals infected at different ages: 5, and 9 weeks to investigate the impact of the physiological development of animals, as well as their different levels of residual MDA, on virus excretion and immunological responses developed after an H1N1 infection*) |  | + |  |  | + | Prevent infection, determine duration of immunity, disease prevention |
| Genzow, 2018 | Efficacy of vaccination (LAIV) in piglets with MDI on viral detection and shedding.  (*The aim of this study was to investigate whether vaccination with this LAIV would reduce viral shedding (duration and incidence) in newborn piglets with or without maternally derived antibodies (MDA)*). | + | + |  |  |  | Prevent infection |

Data was collected tangentially during the systematic review of influenza challenge trials in piglets with maternally immunity derived from vaccinated sows (N= 16) [23].†IVR = The Influenza Vaccine Research and Development (R&D) Roadmap[52]; Research primary objective as stated by author (text in *italics* is as cited in introduction section of the primary study publication); R&D issues 1-5 = identified critical areas of lacking in immunologic understanding limiting the pace and direction of vaccine research as outlined in IVR Roadmap [52]; issue 1 = differences in responses to infection versus vaccination; issue 2 = immune factors and immune mechanisms required to induce broad and durable immunity; issue 3 = the role and drivers of mucosal immunity; issue 4 = the role of imprinting (first exposure) and of repeated exposures on response to vaccination; issue 5 = the role of T-cell responses.

*References:

Allerson, M., Deen, J., Detmer, S. E., Gramer, M. R., Joo, H. S., Romagosa, A., & Torremorell, M. (2013). The impact of maternally derived immunity on influenza A virus transmission in neonatal pig populations. *Vaccine*, *31*(3), 500–505. https://doi.org/10.1016/j.vaccine.2012.11.023

Bosworth, B., Erdman, M. M., Stine, D. L., Harris, I., Irwin, C., Jens, M., Loynachan, A., Kamrud, K., & Harris, D. L. (2010). Replicon particle vaccine protects swine against influenza. *Comparative Immunology, Microbiology and Infectious Diseases*, *33*(6), 99–103. https://doi.org/10.1016/j.cimid.2010.05.002

Cador, C., Rose, N., Willem, L., & Andraud, M. (2016). Maternally derived immunity extends swine influenza A virus persistence within farrow-to-finish pig farms: Insights from a stochastic event-driven metapopulation model. *PLoS ONE*, *11*(9), 1–16. https://doi.org/10.1371/journal.pone.0163672

Deblanc, C., Hervé, S., Gorin, S., Cador, C., Andraud, M., Quéguiner, S., Barbier, N., Paboeuf, F., Rose, N., & Simon, G. (2018). Maternally-derived antibodies do not inhibit swine influenza virus replication in piglets but decrease excreted virus infectivity and impair post-infectious immune responses. *Veterinary Microbiology*, *216*(January), 142–152. https://doi.org/10.1016/j.vetmic.2018.01.019

Genzow, M., Goodell, C., Kaiser, T. J., Johnson, W., & Eichmeyer, M. (2018). Live attenuated influenza virus vaccine reduces virus shedding of newborn piglets in the presence of maternal antibody. *Influenza and Other Respiratory Viruses*, *12*(3), 353–359. https://doi.org/10.1111/irv.12531

Kitikoon, P., Gauger, P. C., Anderson, T. K., Culhane, M. R., Swenson, S., Loving, C. L., Perez, D. R., & Vincent, A. L. (2013). Swine influenza virus vaccine serologic cross-reactivity to contemporary US swine H3N2 and efficacy in pigs infected with an H3N2 similar to 2011-2012 H3N2v. *Influenza and Other Respiratory Viruses*, *7*(SUPPL.4), 32–41. https://doi.org/10.1111/irv.12189

Kitikoon, P., Nilubol, D., Erickson, B. J., Janke, B. H., Hoover, T. C., Sornsen, S. A., & Thacker, E. L. (2006). The immune response and maternal antibody interference to a heterologous H1N1 swine influenza virus infection following vaccination. *Veterinary Immunology and Immunopathology*, *112*(3–4), 117–128. https://doi.org/10.1016/j.vetimm.2006.02.008

Loving, C. L., Brockmeier, S. L., Vincent, A. L., Gauger, P. C., Zanella, E. L., Lager, K. M., & Kehrli, M. E. (2014). Cross-fostering to prevent maternal cell transfer did not prevent vaccine-associated enhanced respiratory disease that occurred following heterologous influenza challenge of pigs vaccinated in the presence of maternal immunity. *Viral Immunology*, *27*(7), 334–342. https://doi.org/10.1089/vim.2014.0034

Pyo, H. M., Hlasny, M., & Zhou, Y. (2015). Influence of maternally-derived antibodies on live attenuated influenza vaccine efficacy in pigs. *Vaccine*, *33*(31), 3667–3672. https://doi.org/10.1016/j.vaccine.2015.06.044

Rajao, D. S., Sandbulte, M. R., Gauger, P. C., Kitikoon, P., Platt, R., Roth, J. A., Perez, D. R., Loving, C. L., & Vincent, A. L. (2016). Heterologous challenge in the presence of maternally-derived antibodies results in vaccine-associated enhanced respiratory disease in weaned piglets. *Virology*, *491*, 79–88. https://doi.org/10.1016/j.virol.2016.01.015

Sandbulte, M. R., Platt, R., Roth, J. A., Henningson, J. N., Gibson, K. A., Rajão, D. S., Loving, C. L., & Vincent, A. L. (2014). Divergent immune responses and disease outcomes in piglets immunized with inactivated and attenuated H3N2 swine influenza vaccines in the presence of maternally-derived antibodies. *Virology*, *464–465*(1), 45–54. https://doi.org/10.1016/j.virol.2014.06.027

Vincent, A. L., Ma, W., Lager, K. M., Richt, J. A., Janke, B. H., Sandbulte, M. R., Gauger, P. C., Loving, C. L., Webby, R. J., & Garcia-Sastre, A. (2012). Live Attenuated Influenza Vaccine Provides Superior Protection from Heterologous Infection in Pigs with Maternal Antibodies without Inducing Vaccine-Associated Enhanced Respiratory Disease. *Journal of Virology*, *86*(19), 10597–10605. https://doi.org/10.1128/jvi.01439-12


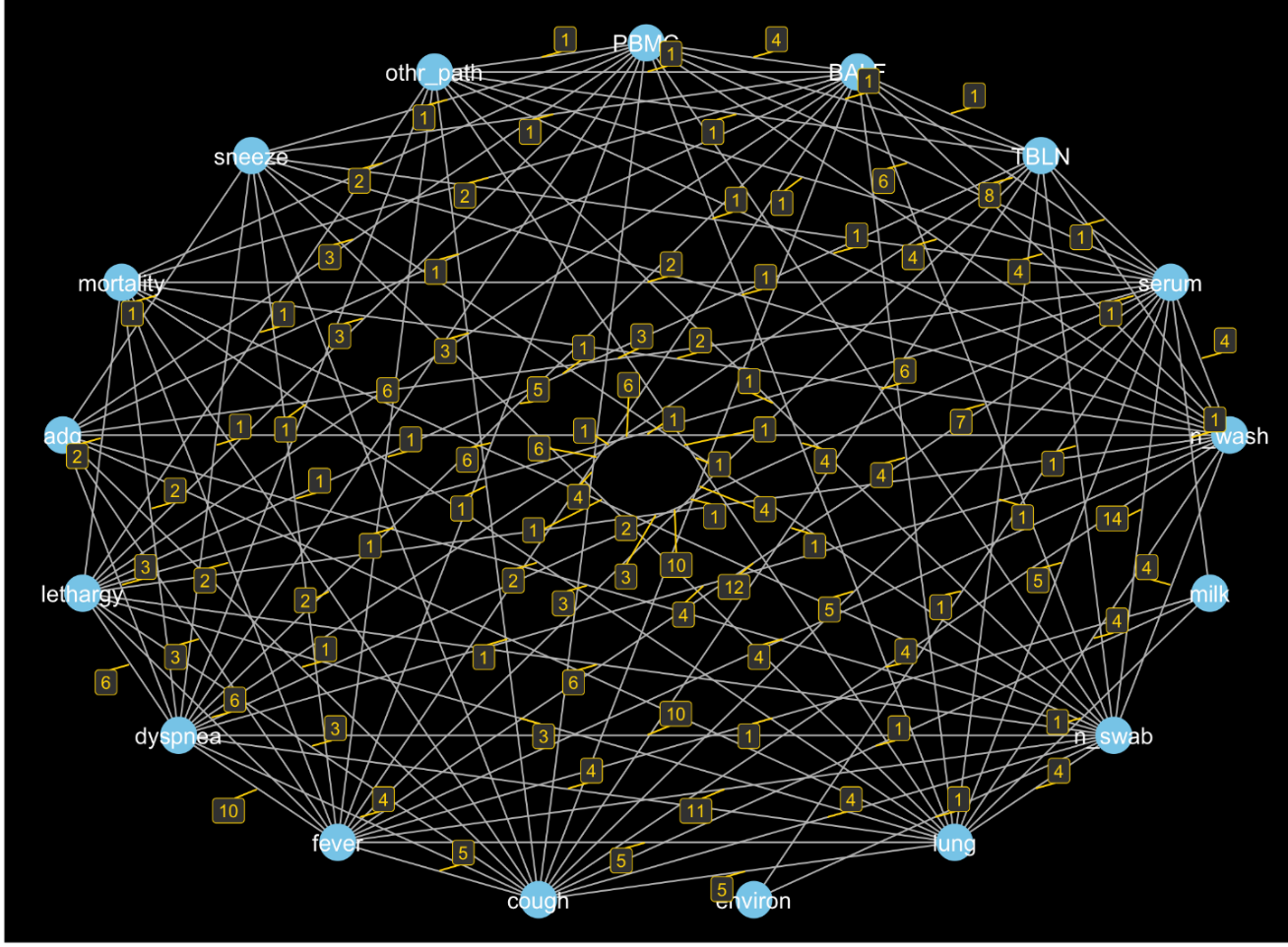


**Supplementary Figure 1. Network analysis of samples collected across 16 studies of MDI protection of swine offspring against influenza challenge.**

Generated using data collected tangentially during the systematic review of influenza challenge trial in piglets with maternally derived immunity from vaccinated sows (N= 16) [23].Blue circles (nodes) = sample type collected in the study; numbers assigned each grey line (edge) = number of studies jointly reporting collection of both sample types; Greater than 10 connections between nodes (i.e. grey lines as edges) are identified by white arrows, frequency of 10 connections between two nodes are identified by blue arrows; PBMN = peripheral blood mononuclear cells (from whole blood); BALF = bronchoaveolar lavage fluids;TBLN = tracheobronchial lymph nodes; n_wash = nasal wash; n_swab = nasal swab; environ = envirnmenatl samples (e.g. air, surfaces); adg = average daily gain, othr_path = tracheal mucosal samples.


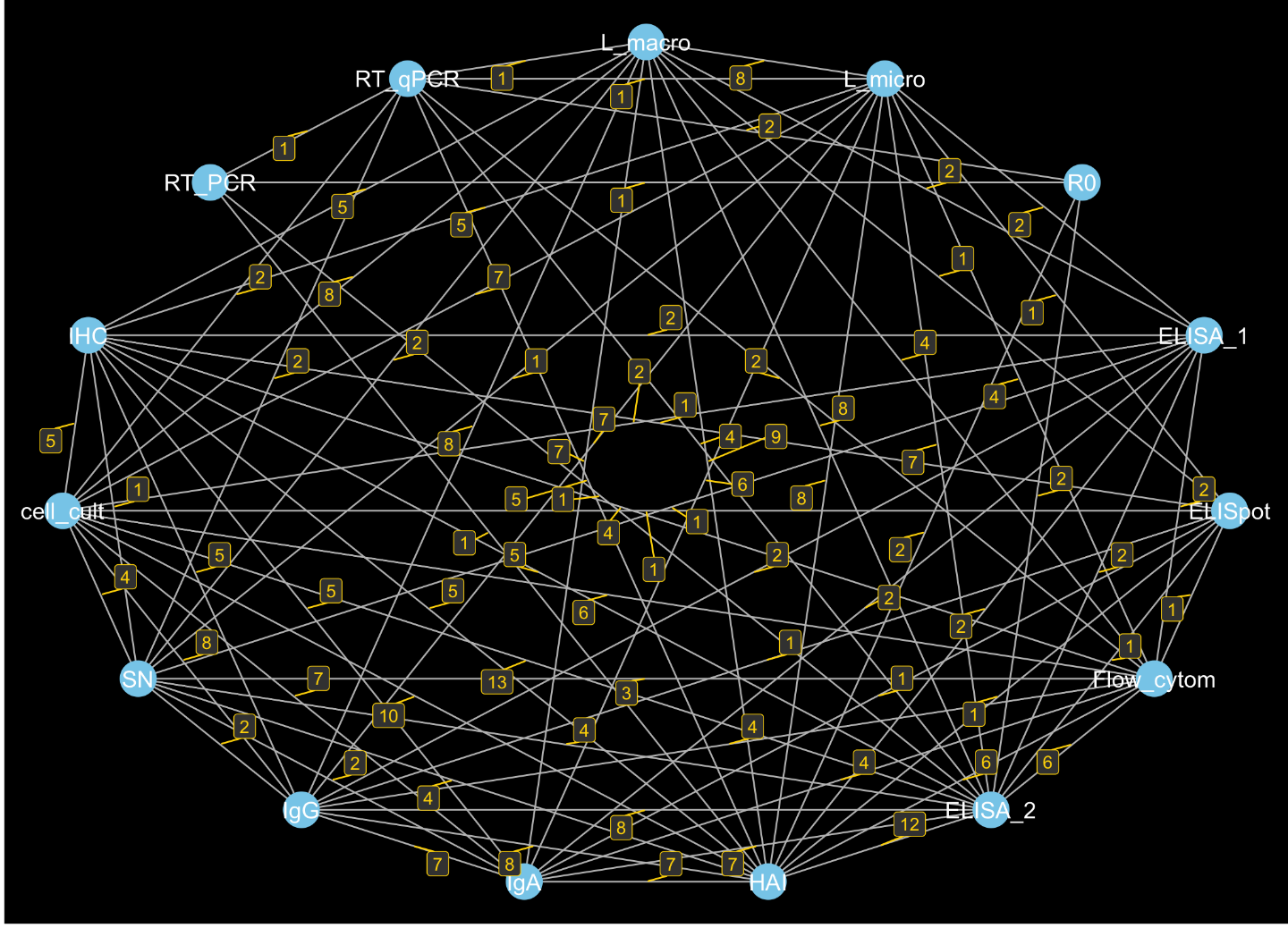


**Supplementary Figure 2. Network analysis of assays employed across 16 studies of MDI protection of swine offspring against influenza challenge.**

Generated using data collected tangentially during the systematic review of influenza challenge trial in piglets with maternally derived immunity from vaccinated sows (N= 16) [23];blue circles (nodes) = assay employed in the study; numbers assigned each grey line (edge) = number of studies jointly reporting use of both assays; most frequent connections between nodes (i.e. grey lines as edges) are identified by white arrows and next highest frequency connections by blue arrows. Assays: HAI = hemaglutinin inhibition assay; ELISA = enzyme linked immunosorbant assay; ELISA _1 = Multiplex ELISA for cytokines; ELISA_2 = detection of immunoglobulins (non-isotype specified); IgG = Assay for detection of isotype IgG; IgA = assay for detection of isotype IgA anti-IAV-S immunoglobulins; L_macro= macroscopic lung assessed; L_micro = microscopic lung assessed; R0 = R-naught (basic reproductive number) ; ELISpot = ELISpot assay for IFN-gamma secreting cells: Flow_cytom = Multi-parameter flow cytometer (T-cell proliferation assay measuring staining for CD4+, CD8+, CD3+ , γδ TCR+ PBMN cell populations (proliferation), and/ or measuring up-regulation of T-cell markers as indicators of T-cell priming (e.g. CD25,IFN-gamma, IL-10)); cell_cult = virus isolation via growth in cultured cells; HAI = Hemagglutination inhibition assay; SN = serum neutralization assay; IHC = immunohistochemistry; RT_PCR = reverse transcriptase polymerase chain reaction; RT_qPCR = quantitative (or real time) reverse transcriptase polymerase chain reaction.

**Supplementary Figure** 3**. Frequency of reported outcomes by sample types collected in primary research investigating MDI protection of swine offspring against influenza virus challenge (N=16).**

Data was collected tangentially during the systematic review of influenza challenge trial in piglets with maternally derived immunity from vaccinated sows (N= 16)[23]. Y-axis = reported outcomes measured (Ig titres = immunoglobulin titres; Virus detection = detection via RT-PCR or virus isolation; virus quantification = virus titres extrapolated from PCR measurements or from virus isolation methods; lung lesion = observed macro or microscopic evaluation; CMI = all non-immunoglobulin immune response including cell mediated immunity; Other = 7 studies reported pathology consistent with VAERD (vaccine associated enhanced respiratory disease - an outcome determined based on measure of lung pathology and clinical presentation of respiratory distress), 4 studies reported outcomes of transmission, 3 studies reported tracheal epithelial pathology); Total = the total number of studies reporting the corresponding Y-axis outcome; X-axis = sample types collected; numbers assigned to each bar equals the total number of studies reporting collection of the corresponding sample type on the X-axis; PBMN = peripheral blood mononuclear cells (from whole blood); BALF = bronchoalveolar lavage fluids; TBLN = tracheobronchial lymph nodes; environ = environmental samples (e.g. air, surfaces).

**Supplementary Figure** 4**. Study assays employed by outcomes measured for 16 studies of MDI protection of swine offspring against influenza challenge.**

Data was collected tangentially during the systematic review of influenza challenge trial in piglets with maternally derived immunity from vaccinated sows (N= 16) [23]. Y-axis = assays employed, X-axis = outcomes measured. Grey bars show the number of studies where the assay was used to measure each corresponding outcome. Assays: L _macro = macroscopic lung assessed; L_micro = microscopic lung assessed; R0 = R-naught (basic reproductive number); ELISA_cyto = Multiplex ELISA for cytokines; ELISpot = ELISpot assay for IFN-gamma secreting cells: Flow_cytom = Multi-parameter flow cytometer (T-cell proliferation assay measuring staining for CD4+, CD8+, CD3+ , γδ TCR+ PBMN cell populations (proliferation), and/ or measuring up-regulation of T-cell markers as indicators of T-cell priming (e.g. CD25,IFN-gamma, IL-10)); cell_cult = virus isolation via growth in cultured cells; ELISA_ab = ELISA for detection of anti-IAV-S antibodies (non-isotype specified); HAI = Hemagglutination inhibition assay; IgA = ELISA specifically for detection of IgA; IgG = ELISA specifically for detection of IgG; SN = serum neutralization assay; IHC = immunohistochemistry; RT_PCR = reverse transcriptase polymerase chain reaction; RT_qPCR = quantitative (or real time); RT = PCR. Outcomes: Antibody = immunoglobulin immune response; Virus detection = identification of virus present via RT-PCR or virus isolation; virus quantification = virus titres from samples using PCR or virus isolation methods; lung lesion = macro/ microscopic evaluation; CMI = all non-immunoglobulin immune response including cell mediated immunity; VAERD = vaccine associated enhanced respiratory disease an outcome determined based on measure of lung pathology and clinical presentation of respiratory distress. Transmission = 2 studies measuring transmission as an outcome using R0 as the method [assay] of measure. Outcomes for clinical signs not included (see Supplementary Figure 5).

**Supplementary Figure** 5**. Frequency of studies reporting clinical signs, by type, in primary research investigating MDI protection of swine offspring against influenza virus challenge (N=16).**

Data was collected tangentially during the systematic review of influenza challenge trial in piglets with maternally derived immunity from vaccinated sows (N= 16) [23]. MDI = maternally derived immunity from vaccination of sows against influenza; Total = the total number of studies reporting clinical signs either as a standalone outcome or as a composite clinical score; ADG = average daily gain; numbers on top of bars are the number of studies reporting measure of the clinical sign.

**Bench-top (Phase 0)** *(in vitro, ex vivo)*

“Proof of concept”

**Exposure Trials (Phase II and IIb)**

Validate CoPs† and endpoints

Determine Immunogenicity &

Dose range/ vaccine schedule

(**IIb – challenge trials or natural exposure‡**)

Efficacy

Same species challenge sufficient for licencing (veterinary vaccines only)

Investigate vaccine resistance (veterinary research)

**Phase IV Post-marketing Trials and Observational Studies**

**(Natural exposure)**

Determine Effectiveness (field efficacy)

Safety over time

Vaccine adverse events

**No Exposure Trials (Phase I)**

Investigate Correlates of Protection†(CoP) Safety

Investigate possible trial endpoints

**Phase III trials (natural exposure)**

Efficacy in target population (pivotal studies = sufficient for licensing)

Safety and tolerability

Veterinary vaccines

Human vaccines

Required for approval for veterinary market

Required for approval for human market

**Supplementary Figure** 6**. Phases of clinical trials and phase- specific endpoints for licensing commercial vaccines for human versus veterinary markets.**

Adopted from Weir (2016), WHO (2004), and Knight-Jones (2014) [42,43,45]; †Immune correlates of protection (CoP) = indirect measures of vaccine effects (e.g. immunoglobulin titres, cytokine or cell mediated responses) and are validated through correlation with direct measures (e.g. infection or clinical disease); ‡Challenge virus exposure = researcher controlled purposeful exposure of study units to pathogen; Natural virus exposure = study units exposed to pathogen naturally; licensed commercial vaccines = openly available for market use as per label claims; Experimental vaccines = not approved for market use; Autogenous vaccines = conditionally approved for defined market use with veterinary oversight (i.e. not open market use).
